# Sarcasm

**Published:** 1893-01

**Authors:** 


### Sarcasm.

A Springfield mother calls her unweaned but teething baby Sarcasm, because he's so biting.

